# Transfer learning with Bayesian optimization for colorectal cancer histopathology classification

**DOI:** 10.1186/s12880-026-02149-x

**Published:** 2026-01-12

**Authors:** Houda Saif ALGhafri, Chia S. Lim

**Affiliations:** 1 Department of Computing and Information Sciences, University of Technology and Applied Sciences, Muscat, 133 Sultanate of Oman; 2 Graduate School of Technology, Asia Pacific University of Technology and Innovation, Kuala Lumpur, 57000 Malaysia

**Keywords:** Transfer learning, Bayesian optimization, Colorectal cancer, Histopathology images

## Abstract

**Background:**

Automated colorectal cancer (CRC) histopathology classification remains challenging due to variations in datasets, staining conditions, and tissue morphology across institutions. Many prior studies apply standard CNN architectures with fixed hyperparameters, leaving limited examination of how model choice and optimization strategies affect performance robustness across heterogeneous CRC data.

**Methods:**

We evaluate eight transfer learning models on three-class CRC datasets and propose CRC-BayTune, applying Bayesian optimization to tune key training parameters, including learning rate, batch size, with fine-tuning depth. All models are assessed in patch-level experimental settings, and statistical significance is quantified using Friedman tests, repeated-measures ANOVA, and post hoc analyses. Robustness is assessed by introducing controlled Gaussian noise perturbations. Grad-CAM provides qualitative visual explanations by highlighting regions that contribute to model predictions.

**Results:**

DenseNet201, InceptionV3, InceptionResNetV2, and ResNet50V2 achieved the highest median MCC values of 0.984, 0.982, 0.975, and 0.983, respectively. Statistical analysis confirms that both model architecture ($\textrm{p}=0.059$, Friedman) and hyperparameter configuration ($\textrm{p}=0.027$, RM-ANOVA) significantly affect performance. Models with deeper feature hierarchies demonstrated more stable convergence and smaller accuracy degradation under noise.

**Conclusion:**

The results show that systematic hyperparameter tuning can improve the training stability and classification performance of standard CNN models compared with fixed configurations in CRC histopathology tasks. The findings underscore that model performance in this setting is sensitive to choices such as learning rate, batch size, and fine-tuning depth, and that evaluating these factors explicitly can support more reliable use of deep learning models in computational pathology.

## Background

Colorectal cancer is the third most common cancer globally, with approximately 1.9 million new cases and 900,000 deaths [1]. Accurate diagnosis is critical for treatment planning and prognosis, with hematoxylin and eosin (H&E) staining remaining the gold standard for assessing tissue abnormalities and invasion depth [2]. However, manual examination is time-consuming, and a shortage of pathologists contributes to delays. Whole slide imaging (WSI) enables digital conversion of glass slides into high-resolution virtual images [3], but the growing volume of pathology data places an additional burden on limited expert resources [4]. Convolutional neural networks (CNNs) can automatically extract features directly from training images [5, 6], offering potential to improve efficiency and precision in CRC histopathology. Yet, high accuracy typically requires deep architectures and large, well-annotated datasets, which remain challenging to obtain.

Image classification is essential for diagnosing CRC histopathology images, with deep neural networks (DNNs), particularly CNNs, widely used for this purpose [7]. Compared to shallow networks, such as single-hidden-layer models [8], and manual microscopic examination, CNNs automatically learn complex features and often outperform conventional approaches on large datasets [9, 10]. Traditional methods that rely on handcrafted features struggle to capture intricate tissue patterns, thereby limiting diagnostic accuracy [10]. The performance of DNNs depends heavily on hyperparameter selection, which often requires extensive manual tuning [11]. In this study, we employ Bayesian optimization to systematically identify hyperparameters, such as learning rate and batch size, that minimize the loss function and improve model accuracy [12].

In this study, we investigate how key hyperparameters, including learning rate and batch size, along with depth fine-tuning, affect the robustness and generalizability of deep learning models for CRC histopathology classification. To address the limitations of fixed or manually selected hyperparameters, we apply Bayesian optimization to automatically identify configurations that minimize training loss and improve accuracy. This approach, referred to as CRC-BayTune, integrates Bayesian optimization with standard transfer learning architectures to provide a systematic and reproducible tuning strategy across multiple CRC datasets. In line with recent advances in artificial intelligence, such as pixel-level necrosis quantification in histopathology [13] and radiomics-based mutation prediction from CT imaging [14], our study focuses on fine-grained classification of mucosa, stroma, and lymph tissues to support microenvironment-aware analysis and more stable model development. The main contributions of our study are as follows:

1. We perform a controlled comparison of eight commonly used convolutional neural network architectures on CRC histopathology, providing quantitative evidence on performance, computational cost, and cross-dataset consistency, an evaluation that is only partially addressed in prior CRC studies.
2. We implement a transparent Bayesian optimization workflow for tuning learning rate, batch size, and fine-tuning depth, offering a reproducible alternative to the fixed or manually selected hyperparameters that dominate existing CRC classification research.
3. We structure the classification task around three routinely examined tissue components-mucosa, stroma, and lymph, ensuring that evaluations focus on diagnostically meaningful regions commonly analyzed in CRC pathology workflows.
4. We evaluate model stability by introducing controlled Gaussian noise at multiple severity levels, quantifying how prediction reliability changes under realistic perturbations that may arise in digital pathology pipelines.
5. We use Grad-CAM to generate qualitative visual explanations, highlighting regions and comparing them against diagnostically relevant tissue structures.

Convolutional neural networks are widely used in CRC histopathology because they learn discriminative features directly from image data, reducing manual annotation demands and inter-observer variability [7]. Transfer learning with pre-trained models is particularly effective for limited datasets, enabling faster convergence and often superior performance compared to training from scratch [15]. Since hyperparameter selection is critical, as it influences model performance, computational efficiency, and memory usage [16–18], careful selection of hyperparameters, such as learning rate and batch size, is essential for ensuring generalization, avoiding overfitting, and maintaining computational efficiency.

Transfer learning with pre-trained CNNs is widely applied in CRC histopathology. For example [19], benchmarked AlexNet, SqueezeNet, VGGNet, GoogLeNet, and ResNet50 on public CRC datasets. They employed SGD-momentum, RMSProp, and Adam with a fixed learning rate (0.01); ResNet50 reached 94.86% after tuning batch size and epochs. [20] fine-tuned ResNet50 with Adam default settings and a batch size of 10 on public CRC data. [21] evaluated VGG19 on public CRC datasets, reporting an accuracy of 91.2%. [22] applied ResNet50 with Adam to CRC images, achieving a validation accuracy of 99.77%. [23] explored VGG16, Xception, DenseNet121, and InceptionResNetV2 with accuracies of 87.2–97.6%. [24] compared Xception, InceptionResNetV2, DenseNet121, VGG16, and a custom CRC-CNN, reporting accuracy of 93.5–99.2%.

Although deep learning has been widely applied to CRC histopathological image classification, most existing studies, such as [19–21, 24], rely on fixed or manually selected hyperparameters, such as learning rate and batch size, rather than systematic optimization. This limits reproducibility and makes model performance sensitive to configuration choices rather than architectural merit. In addition, performance is typically reported on a single dataset, meaning cross-dataset generalization is rarely evaluated. Model robustness is also insufficiently assessed, despite evidence that noise can degrade generalization and inference reliability [25], while controlled Gaussian perturbations can act as a regularizer and improve performance depending on the noise level [26]. Furthermore, prior CRC classification work seldom incorporates statistical significance testing, and external validation is often absent due to dataset constraints. To address these gaps, our study (i) employs Bayesian optimization to automatically determine learning rate and batch size for multiple pre-trained CNN architectures (DenseNet201, EfficientNetV2S, InceptionV3, InceptionResNetV2, MobileNet, MobileNetV2, NASNetMobile, and ResNet50V2), (ii) evaluates generalization using both public and private CRC histopathology datasets, and (iii) assesses robustness under controlled Gaussian noise perturbations, further complemented by (iv) Grad-CAM for model interpretability. This design allows us to quantify performance differences beyond raw accuracy and to provide a reproducible comparison of transfer learning models in the three-class CRC setting.

## Methods

**Fig. 1:**
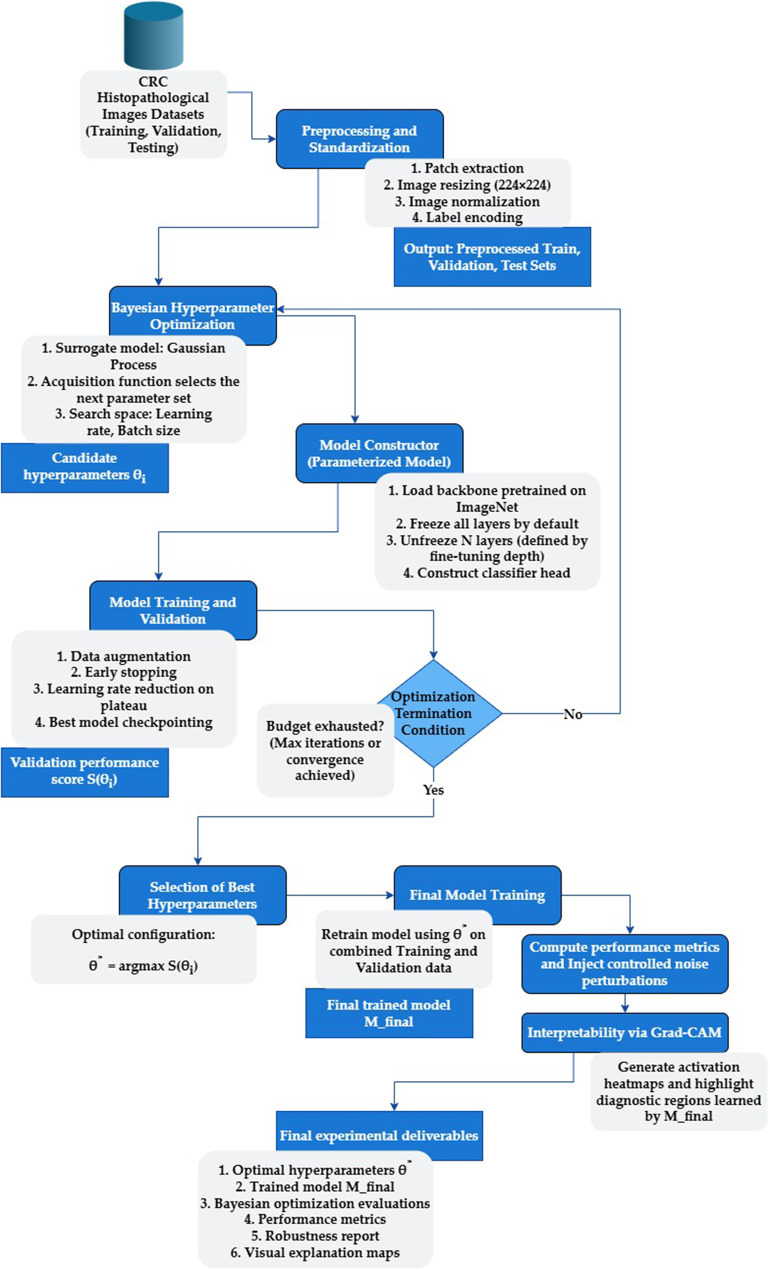
CRC-BayTune pipeline for histopathological image classification, integrating transfer learning with Bayesian-optimized hyperparameters to classify mucosa,stroma, and lymph tissue types

The proposed CRC-BayTune approach performs model development through an iterative Bayesian optimization loop. A search space is defined for learning rate and batch size, and the Bayesian optimizer proposes a candidate hyperparameter set at each iteration. For each proposed configuration, a model is instantiated by loading a pre-trained backbone, freezing the early layers, selectively unfreezing the later layers, and attaching a classification head. The model is then trained using augmented histopathology patches, evaluated on a held-out validation set, and the resulting validation accuracy is returned to the optimizer. This score updates the Gaussian process surrogate, which guides the selection of the next configuration. The process continues for a fixed number of trials, after which the configuration yielding the highest validation performance is selected and retrained before downstream analysis, including robustness testing and interpretability assessment. The complete pipeline is illustrated in Fig. 1, with the optimization procedure detailed in Table 1. The following sections describe each methodological component.

**Table 1:** Algorithm for CRC-BayTune histopathology classification with hyperparameter optimization of the proposed models

**Input:** CRC datasets $D = \{D_{Public1}, D_{Public2}, D_{Private}\}$
**Output:** Optimized classification models $M^{*}$
1: Model Selection:
Define models *M* = {DenseNet201, EfficientV2S, InceptionV3, InceptionResnetV2, MobileNet, MobileNetV2, NASNetMobile, and ResNet50V2}
2. for each dataset $d \in D$:
for each architecture $a \in A$:
Freeze the base layers of $a$
Select fine-tuning depth
Optimize hyperparameters $\theta = \{\text{learning rate}, \text{batch size}\}$ using Bayesian optimization
Apply data augmentation to training images
Train each proposed model $a_d(\theta)$ on augmented training data
end for
Select the best-performing model $M_d^*$ based on validation accuracy
end for
3. Evaluate/ perform statistical tests on the classification performance of the proposed models
4. for each best model $M_d^*$:
Assess robustness under Gaussian noise
Generate Grad-CAM heatmaps for interpretability
5. Return the final set of optimized models, $M^{*}$.

### CRC datasets and preprocessing

Most publicly available CRC histopathology datasets include 8–9 tissue labels [27, 28], many of which exhibit inconsistent annotation across sources. Clinically, key diagnostic decisions, such as identifying dysplasia or evaluating invasion, rely primarily on mucosa, stroma, and lymphoid tissue, which are stable across laboratories and inform AJCC staging. Our study focuses on these three diagnostically relevant tissues, aligning model development with how pathologists reason and improving inter-institutional reproducibility. This pathology-driven selection has not been evaluated in prior CRC deep learning studies, which typically model the complete label set without assessing diagnostic relevance. Colorectal adenocarcinomas, the most common form of CRC, originate in the mucosal epithelium, with progression strongly influenced by stromal responses such as desmoplastic reactions that facilitate invasion and tumor-host interactions [29]. In addition, the accurate localization of lymph nodes and their metastases is crucial for proper staging and can significantly impact clinical management [30].

To improve the diversity of CRC histopathological images, we compiled datasets from two publicly available sources, Dataset 1 consists of 2090 images (Mucosa: 1035, Stroma: 421, Lymph: 634) [28], and Dataset 2 contains 1870 images (Mucosa: 625, Stroma: 625, Lymph: 625) [27]. In addition, we collected a private CRC dataset from the Royal Hospital pathology department in the Sultanate of Oman, in collaboration with expert pathologists, comprising 3717 images (Mucosa: 1480, Stroma: 1119, Lymph Node: 1118), referred to here as Dataset 3. All images were anonymized, and the study was ethically approved; tissue labels were cross-verified by pathologists to ensure reliability. The datasets were then stratified into 70% training and 30% testing subsets. Pixel intensities were normalized to the range [0, 1] to stabilize gradient updates and accelerate convergence during training. All histopathology images were resized to 224 × 224 pixels to ensure a consistent input dimension across different pre-trained models while preserving sufficient spatial detail for tissue structure recognition.

### Transfer learning with pre-trained models

Convolutional neural networks can automatically extract complex image features and leverage transfer learning to improve performance across related datasets [31], addressing the common challenge of limited data [32, 33].

Transfer learning typically freezes pre-trained layers to retain source knowledge and fine-tunes selected layers using backpropagation, with the learning rate controlling weight updates [34]. Retraining the entire network can lead to overfitting on small datasets [35], so selective layer fine-tuning reduces this risk. In our method for CRC histopathology classification, we evaluated eight pre-trained models to determine the best fine-tuning depth, balancing computational efficiency and accuracy to select the final configuration for each model.

We employed a diverse set of pre-trained architectures: DenseNet201 [36], EfficientNetV2S [37], Inception V3 [38], InceptionResNetV2 [39], MobileNet [40], MobileNetV2 [41], NASNetMobile [42], and ResNet50V2 [43] to extract features from CRC histopathology across multiple datasets. DenseNet201 was chosen for its densely connected architecture, which facilitates feature reuse and improves gradient flow, making it especially effective for fine-grained texture analysis. Inception-based models were incorporated due to their multi-scale convolutional pathways, which allow for the efficient extraction of heterogeneous tissue structures. MobileNet, MobileNetV2, and NASNetMobile represent lightweight and efficient designs that aim to reduce computational cost while maintaining competitive accuracy. EfficientNetV2S, designed with compound scaling for balanced accuracy, latency trade-offs, was evaluated to assess the transferability of its scaling principles to CRC histopathology. ResNet50V2 was incorporated as a well-established deep residual baseline for comparison. Together, these models enabled us to evaluate performance across architectures that varied in depth and computational complexity.

### Hyperparameter optimization strategy

Deep learning performance depends critically on properly selected hyperparameters, which must be set prior to training and influence accuracy, generalization, and robustness [44, 45]. Hyperparameter tuning systematically explores combinations to optimize a target metric, as no fixed selection formula exists [46].

#### Bayesian optimization

Hyperparameter optimization is crucial for achieving reliable performance in deep learning; however, it remains computationally challenging in high-dimensional spaces, such as those encountered in medical imaging. Traditional approaches, such as grid search and random search, are straightforward but inefficient, often requiring intensive resources [47–49]. Bayesian optimization provides a principled alternative, iteratively updating a surrogate model, typically a Gaussian Process, using probabilistic modeling to balance exploration of new hyperparameter regions with exploitation of promising ones, thereby converging to optimal configurations with fewer evaluations [46, 50, 51]. Various hyperparameter settings of transfer learning models have not yet been exploited for classifying CRC histopathological images with high performance. In their study, while Adam adaptively tuned the learning rate, they observed that high learning rates with large batch sizes yielded better accuracy, whereas lower learning rates with smaller batch sizes led to improved performance [49]. However, our results, as shown in Table 9, under Bayesian optimization, show otherwise. Batch size and learning rate critically influence model training and performance [44]. Optimizing hyperparameters is especially important in transfer learning to build robust models and ensure high diagnostic accuracy [52].

#### Hyperparameter settings selection

Our study optimizes key hyperparameters, including learning rate and batch size, using Bayesian optimization to streamline tuning and reduce computational overhead. This sequential method models the objective function probabilistically and uses an acquisition function to guide the search toward promising configurations [11, 53]. For CRC histopathology, CNNs automatically extract features but require large datasets; we mitigate data scarcity by leveraging transfer learning with pre-trained models [54]. Combining transfer learning with Bayesian optimization, we assess the impact of hyperparameter choice on training stability, speed, and classification performance [45], aiming to minimize validation loss and maximize predictive accuracy. All experiments in this study were conducted using Python and the TensorFlow framework. The computations were carried out on a system equipped with an NVIDIA GeForce RTX 4060 GPU, a 13th-generation Intel Core i9-11390HX processor, and 32 GB of RAM. Table 2 and 3 present the training and optimization configuration for CRC histopathology classification, yielding the most effective configuration.

**Table 2:** Experimental settings applied to all CRC-BayTune models

Component	Specification
Bayesian optimization	Method: gp_minimize; n_calls $=$ 10; n_initial_points $=$ 5; random_state $=$ 42
Search space	Learning rate $\in \{1\mathrm{e}{-3}, 1\mathrm{e}{-4}, 1\mathrm{e}{-5}, 5\mathrm{e}{-5}, 3\mathrm{e}{-4}\}$; Batch size $\in \{10, 16, 32, 64\}$
Optimizer	ADAM
Epochs	20
Data augmentation	Rotation $\pm 20$, width/height shift $0.1$, shear $0.1$, zoom $0.1$, horizontal flip; fill_mode $=$ “nearest”
Callbacks	EarlyStopping (monitor $=$ validation_loss, patience $=$ 2, restore_best_weights $=$ True); ReduceLROnPlateau (factor $=$ 0.5, patience $=$ 2, min_lr $=$ $1\mathrm{e}{-6}$); ModelCheckpoint (save_best_only $=$ True)

**Table 3:** CRC-BayTune models-specific fine-tuning depth configuration across all CRC datasets

Proposed Model	Fine-tuned Layers (Trainable)	Classifier Head
DenseNet201	Last 40 layers	Dense(512) + Dropout(0.3)
EfficientV2S	Last 200 layers	Dense(1024) + Dropout(0.5)
InceptionV3	Last 150 layers	Dense(1024) + Dropout(0.5)
InceptionResnetV2	Last 80 layers	Dense(1024) + Dropout(0.5)
MobileNet	Last 50 layers	Dense(1024) + Dropout(0.5)
MobileNetV2	Last 80 layers	Dense(1024) + Dropout(0.5)
NASNetMobile	Last 25 layers	Dense(1024) + Dropout(0.5)
ResNet50V2	Last 30 layers	Dense(512) + Dropout(0.3)

### Model interpretability via grad-CAM

To evaluate whether the CRC-BayTune approach relies on biologically meaningful features for classification, we employed Gradient-weighted Class Activation Mapping (Grad-CAM) [55] to generate visual explanations of the predictions made by our proposed models. Analyses were performed on CRC Dataset 3, focusing on three key histological components: mucosa, stroma, and lymph nodes, hypothesized to drive accurate tissue classification. With this approach, we enabled qualitative assessment of the spatial regions prioritized by our proposed models, providing insights into their decision-making process.

## Results

This section presents the experimental evaluation of the proposed models on three CRC histopathology datasets. We first report CRC-BayTune performance with optimized hyperparameters, followed by comparative analyses across datasets. Statistical significance testing, baseline comparisons with fixed hyperparameters, external validation including inference time and model complexity, and interpretability via Grad-CAM are also included. The following sections detail classification performance and robustness across datasets.

### Performance analysis of the proposed models on CRC histopathology datasets

Given the clinical importance of reliable CRC histopathology classification, evaluation was conducted using multiple complementary metrics. Sensitivity ($\displaystyle \frac{\mathrm{TP}}{\mathrm{TP} + \mathrm{FN}}$) quantifies the ability to correctly identify malignant cases, while specificity ($\displaystyle \frac{\mathrm{TN}}{\mathrm{TN} + \mathrm{FP}}$) reflects correct recognition of true negative cases. Precision ($\displaystyle \frac{\mathrm{TP}}{\mathrm{TP} + \mathrm{FP}}$) captures the reliability of positive predictions, and its trade-off with sensitivity is summarized by the F1-score ($\displaystyle \frac{2 \, (\mathrm{Precision} \times \mathrm{Sensitivity})}{\mathrm{Precision} + \mathrm{Sensitivity}}$). To provide a balanced measure under potential class imbalance, the Matthews Correlation Coefficient (MCC) was further computed as $\displaystyle\frac{(\mathrm{TP} \times \mathrm{TN}) - (\mathrm{FP} \times \mathrm{FN})}{\sqrt{(\mathrm{TP} + \mathrm{FP}) (\mathrm{TP} + \mathrm{FN}) (\mathrm{TN} + \mathrm{FP}) (\mathrm{TN} + \mathrm{FN})}}$, where TP, TN, FP, FN denote true positives, true negatives, false positives, and false negatives, respectively.

Table 4 reports the performance of the CRC-BayTune models on the colorectal histopathology datasets, including accuracy, F1-score, and MCC after Bayesian-optimized hyperparameter tuning. DenseNet201 reached the highest patch-level accuracy on Dataset 1 (100%), while EfficientNetV2S showed lower performance on Dataset 2 (71.28%). These results reflect patch-level classification, as Kather 2016 [27], Kather 2019 [28], and the private dataset provide only patch-level annotations. Therefore, the reported metrics represent patch discrimination rather than patient-level diagnostics.

**Table 4:** Evaluation of CRC-BayTune models on three CRC datasets, highlighting differences in classification performance

Proposed Model	**Dataset 1-Accuracy**/ **F1-score/MCC**	**Dataset 2-Accuracy**/ **F1-score/MCC**	**Dataset 3-Accuracy**/ **F1-score/MCC**
DenseNet201	100/ 100/ 100	98.23/ 0.98/ 0.9738	98.75/ 0.99/ 0.9810
EfficientNetV2S	92.68/ 0.91/ 0.8818	71.28/ 0.71/ 0.5852	88.53/ 0.89/ 0.8267
Incepion V3	99.36/ 0.99/ 0.9897	97.87/ 0.98/ 0.9686	99.28/ 0.99/ 0.9892
InceptionResNetV2	99.36/ 0.99/ 0.9898	97.16/ 0.97/ 0.9578	98.57/ 0.99/ 0.9784
MobileNet	99.68/ 1.00/ 0.9949	98.23/ 0.98/ 0.9735	98.03/ 0.98/ 0.9704
MobileNetV2	98.41/ 0.98/ 0.9744	98.23/ 0.98/ 0.9735	99.10/ 0.99/ 0.9864
NASNetMobile	98.41/ 0.98/ 0.9744	95.04/ 0.95/ 0.9259	95.70/ 0.96/ 0.9376
ResNet50V2	99.36/ 0.99/ 0.9897	98.58/ 0.99/ 0.9790	98.75/ 0.99/ 0.9811

MCC values from eight models evaluated on three CRC datasets ($\textrm{N} = 3$ repeated measurements per model) were analyzed using the Friedman test. The test approached significance ($\chi^2(7) = 13.60, \; p = 0.059$), indicating moderate evidence that model choice affected MCC, although not at the conventional threshold ($\alpha = 0.05$). Median MCC scores ranged from 0.827 (EfficientNetV2S) to 0.981 (DenseNet201), with 95% confidence intervals that showed narrow dispersion across models, indicating generally stable performance. Post hoc pairwise comparisons identified several statistically meaningful differences despite the marginal global p-value. EfficientNetV2S showed significantly lower MCC than DenseNet201 ($\textrm{p} = 0.002$), ResNet50V2 ($\textrm{p} = 0.003$), MobileNet ($\textrm{p} = 0.012$), MobileNetV2 ($\textrm{p} = 0.015$), and InceptionResNetV2 ($\textrm{p} = 0.037$). NASNetMobile also underperformed relative to DenseNet201 ($\textrm{p} = 0.008$) and InceptionV3 ($\textrm{p} = 0.037$). No adjusted comparisons showed a significant performance decrease for DenseNet201, InceptionV3, or ResNet50V2, suggesting that these deeper architectures achieved consistently higher MCC across the available data. The Friedman result showed that the global test did not cross the 0.05 threshold, but the pattern of post-hoc outcomes consistently favored deeper models, supporting the conclusion that architectural design had a measurable impact on predictive performance.

Table 5 compares validation accuracy of CRC-BayTune models using fixed hyperparameters (learning rate $=$ 1e-4, batch size $=$ 32) against Bayesian-optimized settings. Across most architectures and datasets, Bayesian optimization consistently improved performance, with gains up to 36.5%, demonstrating the effectiveness of automated hyperparameter tuning.

**Table 5:** Comparison of CRC-BayTune validation accuracy with fixed hyperparameters versus Bayesian optimization. Learning rate fixed at 1e-4 and batch size at 32 for baseline

Model	**Validation Accuracy**/ **(Baseline)**	**Validation Accuracy**/ **Bayesian optimization**	Difference
DenseNet201-Dataset1	0.9925	1.0000	+0.0075
DenseNet201-Dataset2	0.9916	0.9916	0
DenseNet201-Dataset3	0.9957	0.9937	−0.002
EfficientNetV2S-Dataset1	0.9064	0.9513	+0.0449
EfficientNetV2S-Dataset2	0.7322	0.7573	+0.0251
EfficientNetV2S-Dataset3	0.8861	0.9051	+0.019
InceptionV3-Dataset1	0.9963	1.0000	+0.0037
InceptionV3-Dataset2	0.9833	0.9874	+0.0041
InceptionV3-Dataset3	0.9789	0.9895	+0.0106
InceptionResNetV2-Dataset1	0.9962	1.0000	+0.0038
InceptionResNetV2-Dataset2	0.9623	0.9833	+0.021
InceptionResNetV2-Dataset3	0.9873	0.9916	+0.0043
MobileNet-Dataset1	0.9850	1.0000	+0.015
MobileNet-Dataset2	0.9833	0.9833	0
MobileNet-Dataset3	0.9810	0.9873	+0.0063
MobileNetV2-Dataset1	0.9513	0.9963	+0.045
MobileNetV2-Dataset2	0.7364	0.9791	+0.2427
MobileNetV2-Dataset3	0.6245	0.9895	+0.365
NasNetMobile-Dataset1	0.9775	0.9888	+0.0113
NasNetMobile-Dataset2	0.9707	0.9665	−0.0042
NasNetMobile-Dataset3	0.9388	0.9409	+0.0021
ResNet50V2-Dataset1	1.0000	1.0000	0
ResNet50V2-Dataset2	0.9707	0.9833	+0.0126
ResNet50V2-Dataset3	0.9895	0.9916	+0.0021

External validation was performed using two independent datasets: Private Dataset 3 and Public Dataset 2 to assess generalization beyond the source data. Across models, accuracy decreased compared with internal evaluation, confirming the expected domain shift. As shown in Table 6, external accuracy varied by architecture, with the strongest results achieved on Public Dataset 2 (ResNet50V2: 78.03%, InceptionResNetV2: 76.48%, InceptionV3: 75.09%), whereas accuracy decreased more noticeably on the private dataset due to its distinct staining and acquisition characteristics. Inference was efficient for all models, with average per-image latency ranging from 1.45 to 6.48 ms.

**Table 6:** Performance of all models trained on public dataset 1 (internal) and external validation accuracy and inference time of CRC-BayTune models on two independent datasets

Model	External Dataset	Accuracy (%)	Total Inference Time (s)	Average Time per Image (ms)
DenseNet201	Private Dataset 3	52.46	23.3383	6.28
	Public Dataset 2	65.07	10.6805	5.70
EfficientNetV2S	Private Dataset 3	52.46	24.1036	6.48
	Public Dataset 2	53.81	12.0202	6.41
InceptionV3	Private Dataset 3	59.32	8.9971	2.42
	Public Dataset 2	75.09	4.2714	2.28
InceptionResNetV2	Private Dataset 3	55.42	15.3770	4.14
	Public Dataset 2	76.48	11.3921	6.08
MobileNet	Private Dataset 3	48.16	5.4053	1.45
	Public Dataset 2	63.84	2.8450	1.52
MobileNetV2	Private Dataset 3	46.62	5.8871	1.58
	Public Dataset 2	68.43	4.0037	2.14
NasNetMobile	Private Dataset 3	53.97	12.6290	3.40
	Public Dataset 2	68.00	3.9481	2.11
ResNet50V2	Private Dataset 3	52.87	9.0037	2.42
	Public Dataset 2	78.03	6.2542	3.34

Table 7 summarizes the total, trainable, and non-trainable parameters of each CRC-BayTune model, highlighting their relative complexity.

**Table 7:** Model complexity of CRC-BayTune architectures, including total, trainable, and non-trainable parameters

Model	Total Params	Trainable	Non-trainable
DenseNet201	19,307,075	2,396,995	16,910,080
EfficientNetV2S	21,646,179	14,711,619	6,934,560
InceptionV3	23,904,035	18,742,467	5,161,568
InceptionResNetV2	55,913,699	14,546,627	41,367,072
MobileNet	4,281,539	4,125,443	156,096
MobileNetV2	3,572,803	3,382,531	190,272
NasNetMobile	5,355,159	1,255,635	4,099,524
ResNet50V2	24,615,427	15,494,147	9,121,280

Table 8 summarizes the classification performance of our CRC-BayTune models compared with previous studies using [27, 28] datasets. Across both 8-class and 9-class patch-level tasks, DenseNet201, InceptionResNetV2, and MobileNetV2 consistently achieve high accuracy, demonstrating competitive performance relative to published CNN models.

**Table 8:** Benchmark comparison of CRC-BayTune models with published CNN studies on Kather et al. datasets. Evaluation is at the patch-level for 8-class and 9-class tasks

Study	Model	Dataset / Classes	Accuracy (%)
[19]	ResNet50	[27] / 8	94.18
[20]	ResNet50	[28] / 9	97.7
[21]	VGG19	[27] / 8	91.2
[24]	Xception	[27] / 8	88.20
	DenseNet121	[27] / 8	87.20
	VGG16	[27] / 8	90.40
Current Study	DenseNet201	[27] / 8	96.08
	EfficientNetV2S	[27] / 8	61.29
	InceptionV3	[27] / 8	93.57
	InceptionResNetV2	[27] / 8	92.95
	MobileNet	[27] / 8	94.98
	MobileNetV2	[27] / 8	95.92
	NasNetMobile	[27] / 8	88.71
	ResNet50V2	[27] / 8	93.73
Current Study	DenseNet201	[28] / 9	99.45
	EfficientNetV2S	[28] / 9	80.02
	InceptionV3	[28] / 9	99.13
	InceptionResNetV2	[28] / 9	98.80
	MobileNet	[28] / 9	99.67
	MobileNetV2	[28] / 9	98.80
	NasNetMobile	[28] / 9	94.54
	ResNet50V2	[28] / 9	98.58

### Robustness of the proposed models to Gaussian noise for CRC histopathology

Robustness is essential in medical imaging to maintain consistent diagnostic accuracy despite variations in patient data and imaging conditions [56]. Image noise can obscure features, reduce accuracy, and impair generalization [57]. In CRC histopathology, we evaluate model stability using controlled Gaussian noise, providing a standardized and reproducible measure of sensitivity across architectures. This approach isolates the effect of signal degradation without confounding factors such as staining or scanner differences, offering a baseline stress test for comparative robustness assessment.

According to [58], Gaussian noise is a type of additive statistical noise, where each pixel in a noisy image is the sum of its original value and an independent random value drawn from a normal distribution. In our study, the clean test set was first normalized to the range of [0, 1]. For each predefined noise level $\sigma \in \{0.00, 0.05, 0.10, 0.15, 0.20\}$, we generated noise from a zero-mean Gaussian distribution with standard deviation $\sigma$ and added it independently to every pixel in the test images. After adding noise, pixel values were clipped to [0,1] to maintain valid intensities. The noisy images were then input to the trained models, and classification accuracy was measured against the true labels. This experiment evaluates model robustness: well-generalized models should maintain high accuracy at low noise levels, with gradual performance decline as noise increases. CRC models were tested under progressive Gaussian noise, where low levels may enhance robustness, while higher levels are expected to reduce accuracy.

**Fig. 2:**
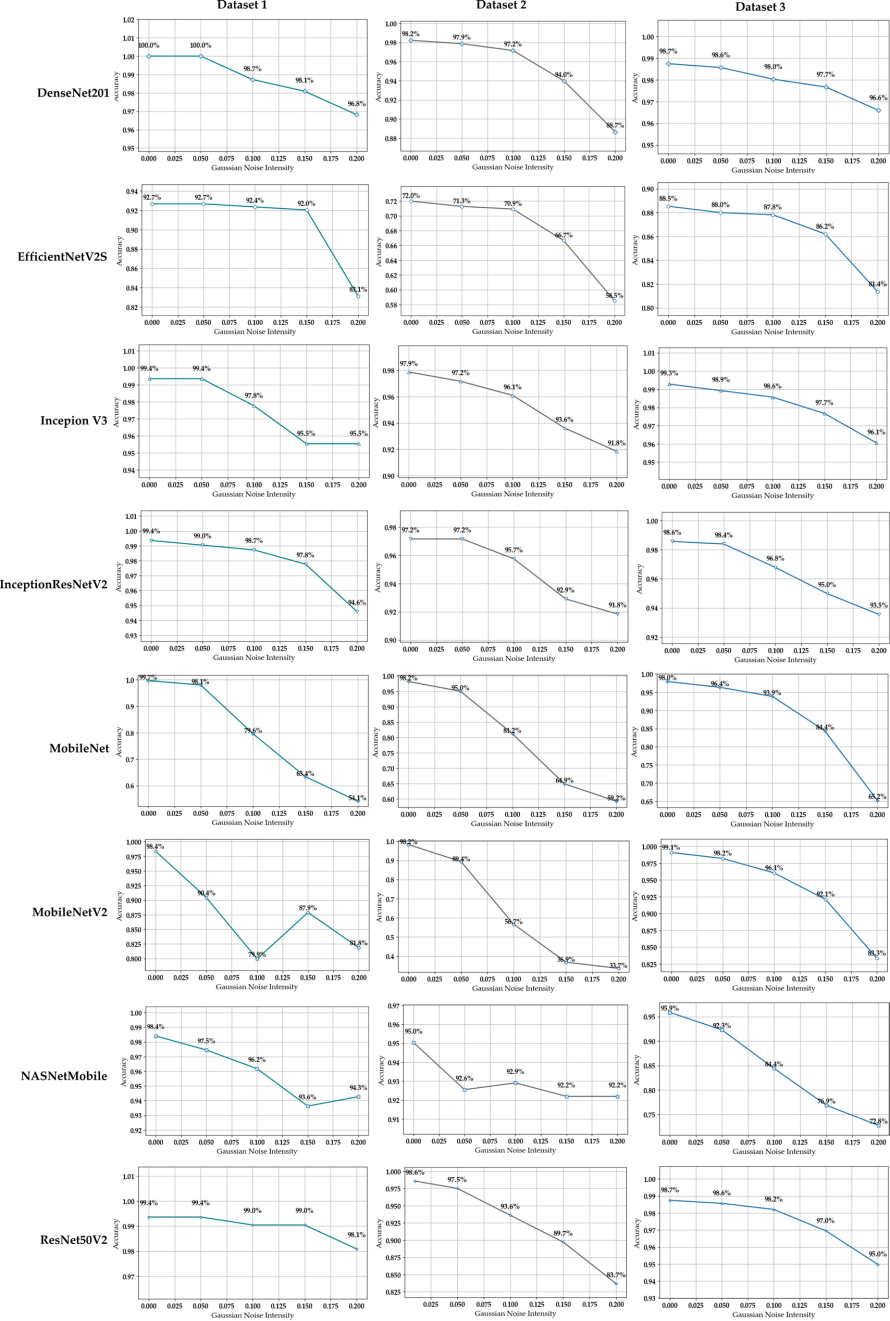
Effect of incremental Gaussian noise on the classification performance of CRC-BayTune models across multiple CRC datasets

The observed experimental results align with the stated hypothesis. Across the models and datasets, accuracy remained relatively stable for noise levels with $$\sigma \le 0.15$$, while higher noise levels $$\sigma > 0.15$$ up to 0.20, led to declines in performance. This pattern was consistent across architectures, with low Gaussian noise maintaining performance and higher noise reducing robustness. CRC-BayTune evaluation results are summarized in (Fig. 2), with detailed metrics provided in Table 9. To assess robustness differences between architectures under noise, we applied a Repeated Measures ANOVA using eight models evaluated on three CRC datasets ($$\textrm{N} = 3$$ repeated measures per model). Accuracy values satisfied normality based on the Shapiro–Wilk test ($$p = 0.088$$–$$1.000$$). The ANOVA revealed a significant overall effect of model choice on accuracy under noise ($$F(7,14) = 3.32, \; p = 0.027, \; \eta^2 = 0.526$$), indicating that 52.6% of the variance in robustness was attributable to architectural differences. Median accuracy ranged from 0.800 to 0.980, with 95% CI [$$0.865, 0.947$$]. Tukey post hoc comparisons did not yield statistically significant pairwise results (all adjusted $$p$$-values $$ > 0.16$$), which is consistent with the limited statistical power associated with only three repeated measurements per model. However, several comparisons showed notable numerical performance gaps; for example, DenseNet201 exceeded MobileNet by 0.146 ($$\textrm{SE} = 0.0304$$, $$t = 4.80$$) and InceptionV3 exceeded MobileNet by 0.142 ($$\textrm{SE} = 0.0264$$, $$t = 5.36$$), although neither difference reached corrected significance. For example, Dataset 2 (Table 4), with a smaller sample size and lower original resolution ($$150\times150$$ pixels, resized to $$224\times 224$$), consistently showed reduced classification accuracy across models. Such effects (Fig. 2) were most evident in EfficientNetV2S, NASNetMobile, and MobileNet variants, which were more sensitive to Gaussian noise perturbations. This suggests that both dataset characteristics (size and resolution) and architectural properties (model depth and parameterization) influence how hyperparameter optimization translates into robustness.

**Table 9:** Robustness scores of CRC-BayTune under Gaussian noise, evaluated using the best hyperparameter settings for each CRC dataset

Model	Dataset	Learning Rate	Batch Size	Robustness
DenseNet201	Dataset 1	$5 \times 10^{-5}$	32	0.988
	Dataset 2	$1 \times 10^{-5}$	10	0.956
	Dataset 3	$1 \times 10^{-4}$	32	0.980
EfficientNetV2S	Dataset 1	$5 \times 10^{-5}$	64	0.913
	Dataset 2	$1 \times 10^{-4}$	10	0.685
	Dataset 3	$1 \times 10^{-4}$	16	0.867
InceptionV3	Dataset 1	$1 \times 10^{-5}$	10	0.975
	Dataset 2	$5 \times 10^{-5}$	32	0.954
	Dataset 3	$1 \times 10^{-3}$	32	0.982
InceptionResNetV2	Dataset 1	$1 \times 10^{-4}$	64	0.981
	Dataset 2	$3 \times 10^{-4}$	16	0.951
	Dataset 3	$1 \times 10^{-3}$	32	0.966
MobileNet	Dataset 1	$5 \times 10^{-5}$	10	0.795
	Dataset 2	$1 \times 10^{-4}$	10	0.8
	Dataset 3	$1 \times 10^{-3}$	10	0.891
MobileNetV2	Dataset 1	$1 \times 10^{-5}$	10	0.871
	Dataset 2	$1 \times 10^{-5}$	10	0.622
	Dataset 3	$1 \times 10^{-4}$	16	0.944
NASNetMobile	Dataset 1	$5 \times 10^{-5}$	16	0.959
	Dataset 2	$1 \times 10^{-5}$	10	0.928
	Dataset 3	$5 \times 10^{-5}$	64	0.845
ResNet50V2	Dataset 1	$5 \times 10^{-5}$	64	0.99
	Dataset 2	$5 \times 10^{-5}$	32	0.93
	Dataset 3	$5 \times 10^{-5}$	32	0.977

As shown in Table 9, across all proposed models applied to CRC datasets, we observe that robustness is not determined by learning rate or batch size in isolation, but by their interaction with model capacity and dataset complexity. High-capacity architectures such as DenseNet201 and ResNet50V2 consistently attained peak robustness in a low learning rate $(\approx 5 \times 10^{-5})$ and moderate-large batch size (32–64), whereas such models as only reached competitive robustness at higher learning rates $(\approx 1 \times 10^{-3},\ 1 \times 10^{-4})$ with small batch sizes. This contrast evidences a reproducible phenomenon: robustness is conditionally predictable when learning rate and batch size are interpreted in the context of architectural depth and dataset variability. Hence, Bayesian optimization systematically identifies these configurations beyond fixed hyperparameters.

**Fig. 3:**
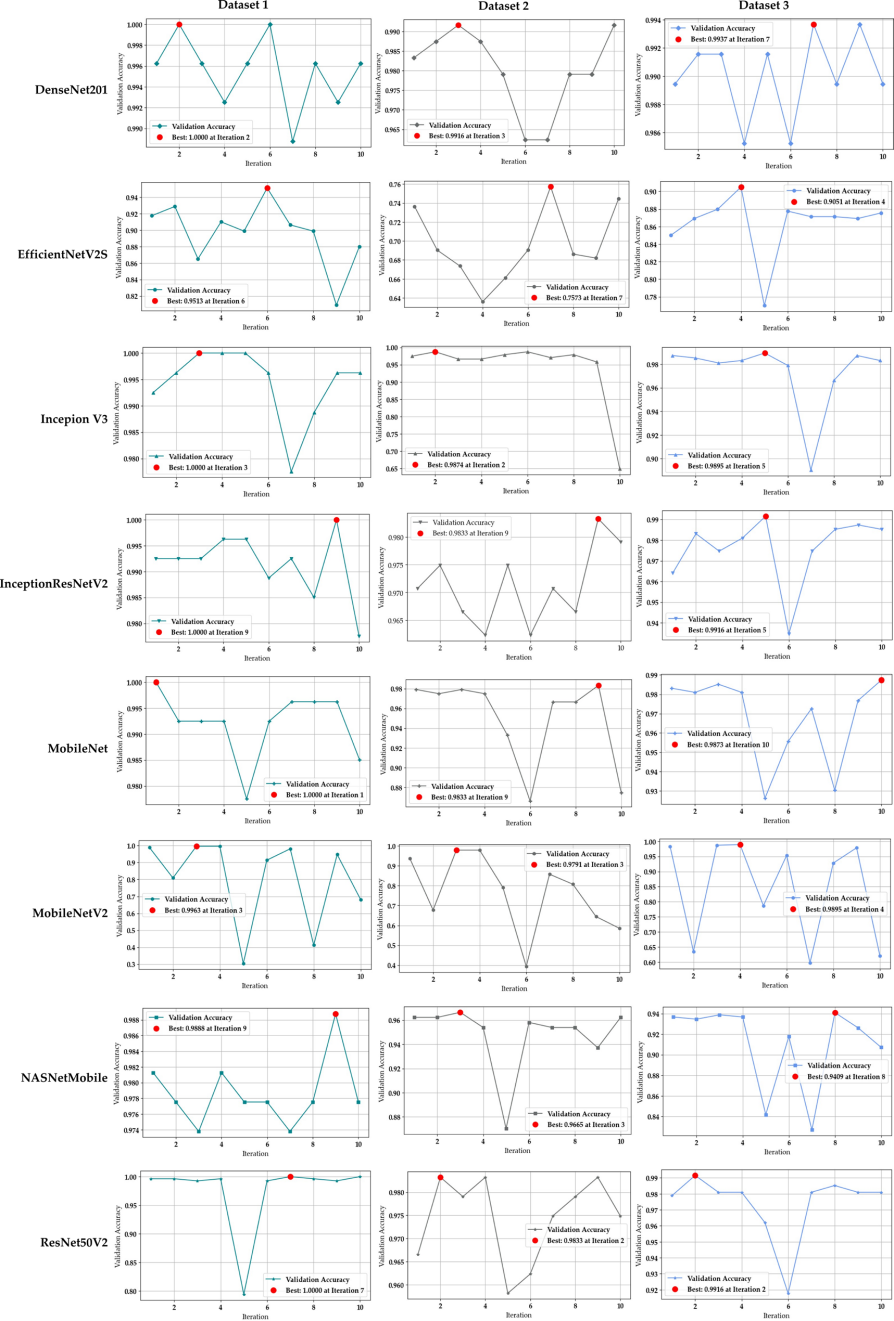
Validation accuracy trends across training iterations for CRC-BayTune models, illustrating convergence behavior

Fig. 3 shows that validation performance stabilizes within the first few iterations, indicating that the low-dimensional search space (learning rate and batch size) was adequately explored with ten optimization calls. While a larger budget would further refine the search, this configuration was sufficient to reach stable optima in our setting. For example, ResNet50V2 in Datasets 1 and 3, DenseNet201 in Dataset 1, and InceptionV3 in Dataset 1 achieved near-optimal validation accuracy, indicating that smooth hyperparameter landscapes were effectively exploited by Bayesian optimization. In contrast, EfficientNetV2S and the MobileNet family exhibited sharp accuracy drops or repeated zigzagging patterns across iterations, reflecting higher sensitivity to unstable or noisy regions of the search space. These results highlight the importance of considering both stability and final accuracy when we optimize CRC histopathology classifiers.

### Grad-CAM visualization

**Fig. 4:**
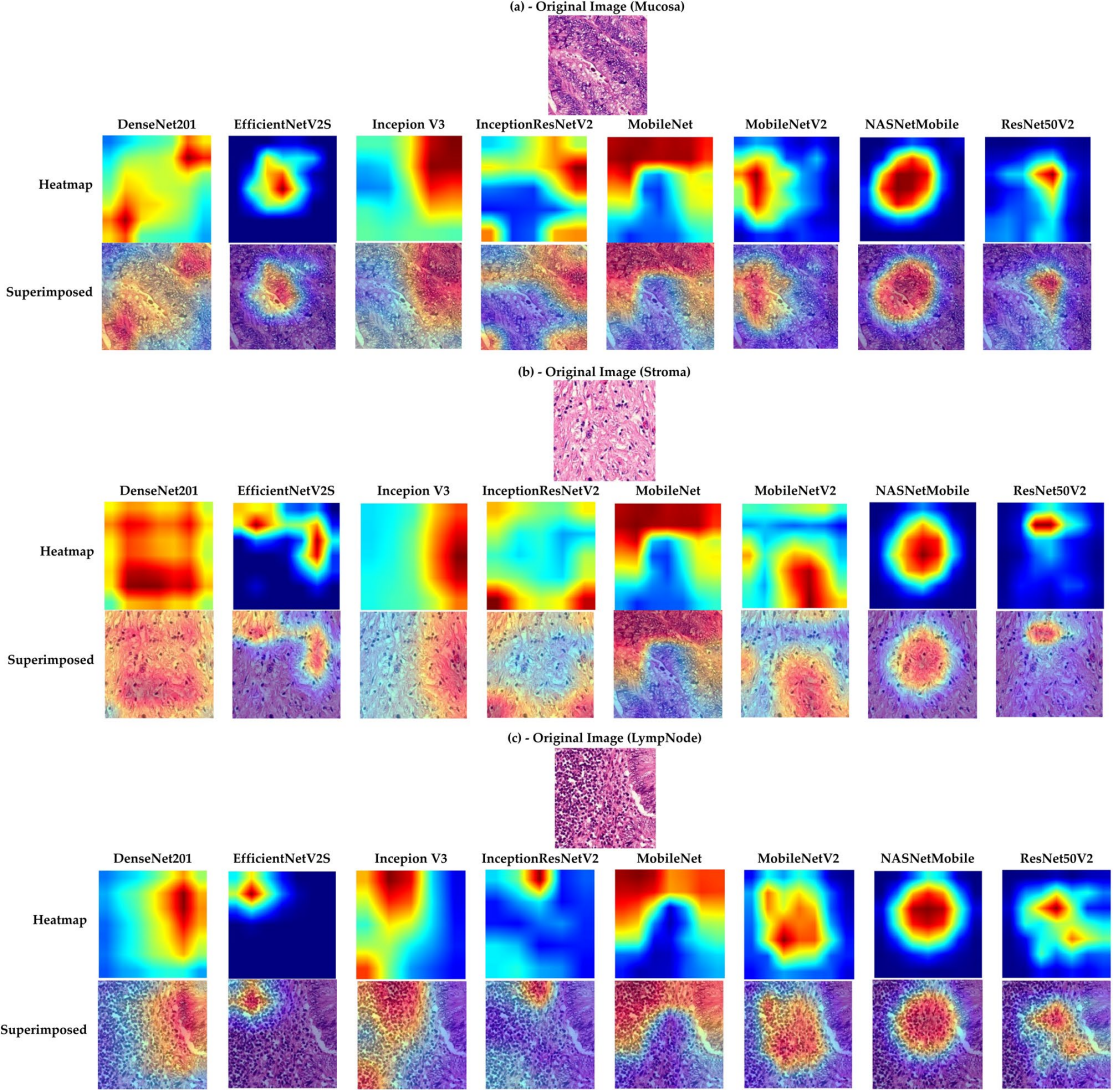
Grad-CAM visualizations of representative CRC dataset 3 samples. (**a**) Mucosa regions highlighting varying levels of malignancy detection across the proposed models. (**b**) Stroma regions showing relative feature emphasis. (**c**) Lymph nodes illustrating the capture of malignancy-related mucosal and lymphatic features by the proposed models

Grad-CAM analysis (Fig. 4) showed model-specific differences in capturing pathological features. In malignant mucosa (a), DenseNet201, InceptionV3, and InceptionResNetV2 consistently localized dysplastic glands, while other models highlighted mucosal regions with fewer malignant glands. In stroma (b), all models identified stroma features, with DenseNet201 showing the most consistent emphasis. For lymph nodes (c) containing malignant mucosa, DenseNet201 and MobileNet localized to metastatic foci, whereas others primarily highlighted nodal tissue. These results indicate that our proposed models, combined with tissue-focused training, influence both predictive accuracy and alignment with pathology-relevant features.

## Discussion

Optimization identifies parameter values that improve model performance [59]. Bayesian optimization achieves this using a Gaussian process surrogate to model the objective function, which is iteratively updated to predict outcomes for unexplored parameters [60, 61]. An acquisition function balances exploration and exploitation to efficiently select new evaluation points [59]. Our results indicate that architecture choice and hyperparameter stability directly influence CRC histopathology classification performance. Models such as DenseNet201, InceptionV3, and ResNet50V2 achieved higher accuracy, F1, and MCC because their deeper feature pipelines and skip connections enabled better representation of heterogeneous tissue structures. Friedman testing confirmed that performance differences across models were statistically significant, demonstrating that architecture selection cannot be treated as interchangeable. Robustness experiments with progressively increasing Gaussian noise showed that some architectures maintained performance, while others degraded earlier, and Repeated Measures ANOVA confirmed these differences across datasets. These outcomes show that systematic hyperparameter tuning is effective in stabilizing training and reducing model sensitivity to tissue variability, supporting more reliable use of CNNs in CRC image analysis.

Through Bayesian optimization, we observe a correlation between architectural design, fine-tuning depth, and convergence stability in CRC histopathology classification. While some of our proposed models stabilized quickly, others, such as EfficientNetV2S and MobileNet, exhibited oscillations or sharp accuracy drops, highlighting that reliable CRC classification requires both stable optimization and robustness to dataset-specific variability. For example, residual and densely connected architectures such as ResNet50V2 (fine-tuned the last 30 layers) and DenseNet201 (last 40 layers) converge reliably, aligning with their design objectives of improved gradient flow and feature reuse. Architecture like NASNetMobile stabilized with minimal fine-tuning (last 25 layers), but its robustness was lower and more sensitive to the shift, dataset-dependent on CRC. In contrast, models with lighter or factorized structures, such as InceptionV3 (last 150 layers), EfficientNetV2S (last 200 layers), and MobileNet/V2 (last 50–80 layers), require broader retraining, reflecting their reliance on deeper adaptation to CRC tissue features. This approach highlights the necessity of architecture-aware tuning to achieve robust CRC histopathology classification via deep learning approaches. The external evaluation highlights how architectural differences and dataset characteristics influence generalization performance. Public Dataset 2 produced higher accuracies across most models, reflecting closer alignment with the development data, whereas Private Dataset 3 introduced greater variability. Inference was computationally efficient across models; future extensions incorporating diverse datasets would allow a more comprehensive assessment of generalization. The comparison confirms that Bayesian optimization provides a systematic advantage over fixed configurations, particularly for models sensitive to hyperparameter choices such as MobileNetV2, highlighting its value for enhancing predictive performance and robustness in CRC histopathology classification. Moreover, model complexity affects both computational cost and practical deployment: larger architectures, such as InceptionResNetV2, offer rich features but higher memory demands, while lightweight models, such as MobileNet, achieve efficiency without heavily compromising capacity. In addition, Grad-CAM visualizations provide qualitative insight into the regions that influence model predictions; however, formal clinical interpretability requires expert pathologist verification. While our high-performing models often focus on morphology consistent with CRC tissue patterns, this alignment has not been quantitatively assessed. Strengthening the clinical relevance of the framework will therefore require evaluation on multi-center datasets, incorporation of pathologist feedback, and testing under broader sources of variability to better reflect real-world diagnostic conditions.

## Conclusions

Our study demonstrates that transfer learning models, optimized with Bayesian hyperparameter tuning, achieve high accuracy and robustness across CRC histopathological datasets, even in the presence of Gaussian noise. Statistical analysis using 95% confidence intervals confirmed consistent performance, indicating reliable classification results. Notably, robustness was influenced not by learning rate or batch size in isolation, but by their interaction with model capacity and dataset complexity, underscoring the value of systematic hyperparameter optimization. Future work should extend evaluation to cross-institutional datasets and broader perturbation scenarios to further validate generalizability.

## Data Availability

The public datasets utilized in this study are accessible via Zenodo (https://zenodo.org/records/1214456 and https://zenodo.org/records/53169). Additional data supporting the findings of this study are available from the corresponding author upon reasonable request.

## Abbreviations

CNNs: Convolutional Neural Networks
CRC: Colorectal Cancer
CRC-BayTune: CRC-Bayesian Tuning
DNNs: Deep Neural Networks
FN: False Negatives
FP: False Positives
Grad-CAM: Gradient-weighted Class Activation Mapping
H&E: Hematoxylin and Eosin
MCC: Matthews Correlation Coefficient
TN: True Negatives
TP: True Positives
WSI: Whole Slide Imaging
